# Supplementary material for: Structure of a 10-23 deoxyribozyme exhibiting a homodimer conformation
Source: Commun Chem. 2023 Jun 10;6:119. doi: 10.1038/s42004-023-00924-3 (PMC10257704; doi:10.1038/s42004-023-00924-3)
Supplement: Supplementary file 1 — Supplementary Information [file 42004_2023_924_MOESM1_ESM.pdf]

Supplementary Figures:

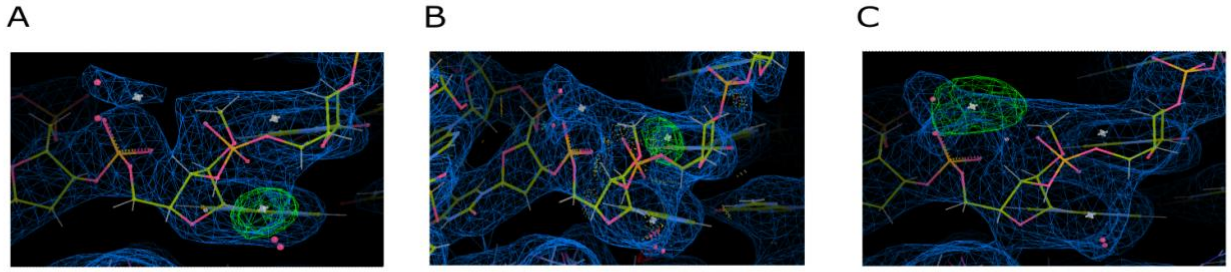

Supplementary Figure 1: Replacement of active site magnesium ions with sodium. Active site magnesium was replaced one by one with sodium and then run through a subsequent round of refinement. The positive peaks indicate the need for more electron density indicating that magnesium is appropriate at these sites. The replacements move from M1-3 (A-C respectively). FoFc σ= 4.0, 2FoFc σ= 1.0

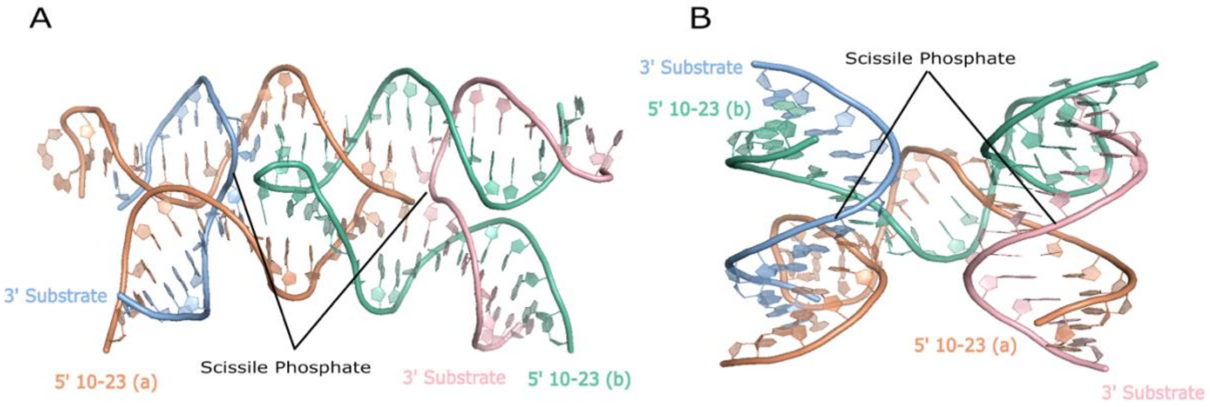

Supplementary Figure 2: Comparison to past 10-23 crystal structure. (A) current 10-23 DNAzyme crystal structure that showcases cis-binding of DNAzyme to substrate and a bend in the substrate strand at the catalytic core. (B) Past crystal structure of the 10-23 DNAzyme with trans-binding of the catalytic core to substrate and a linear substrate with no bending at the scissile phosphate.

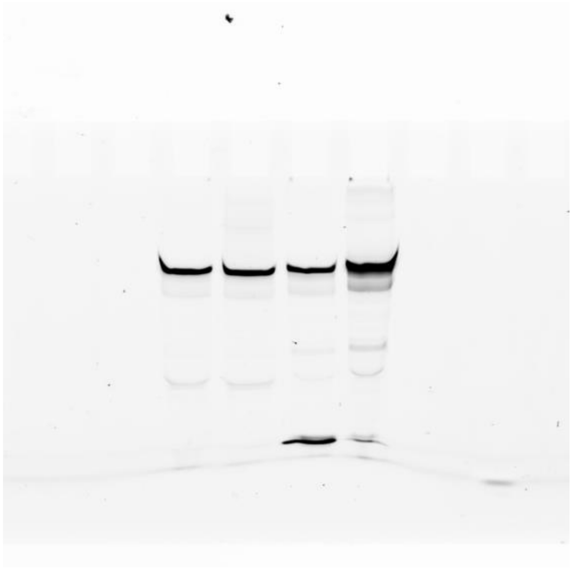

Supplementary Figure 3: Uncropped image of Figure 1B.

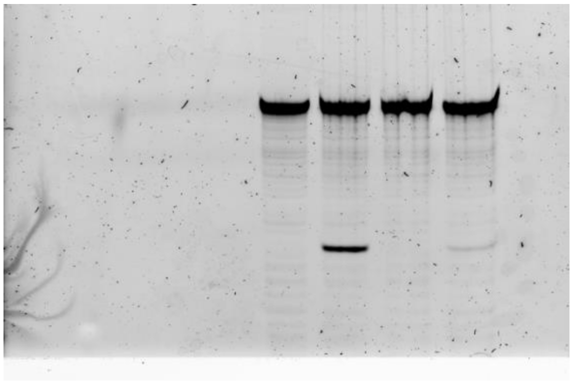

Supplementary Figure 4: Uncropped image of figure 3A.

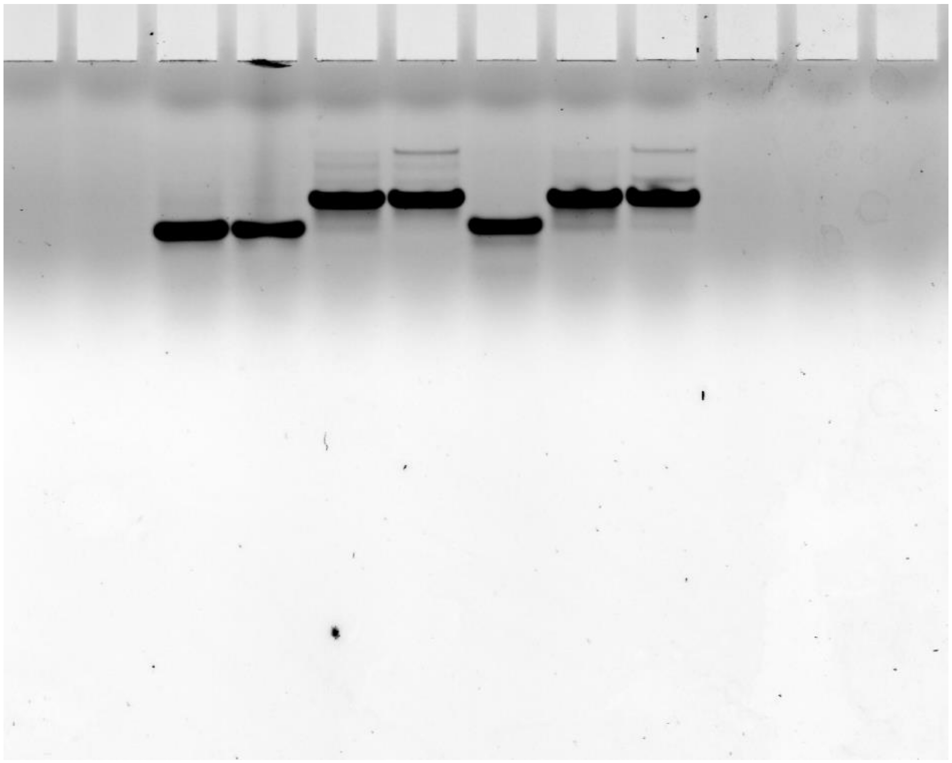

Supplementary Figure 5: Uncropped image of Figure 3B. Additional three lanes the left are repeats of lanes 2-5 in Figure 3B but with the base pair adjacent to the cleavage site being an A-U base pair rather than the G-C base pair used for the structure.

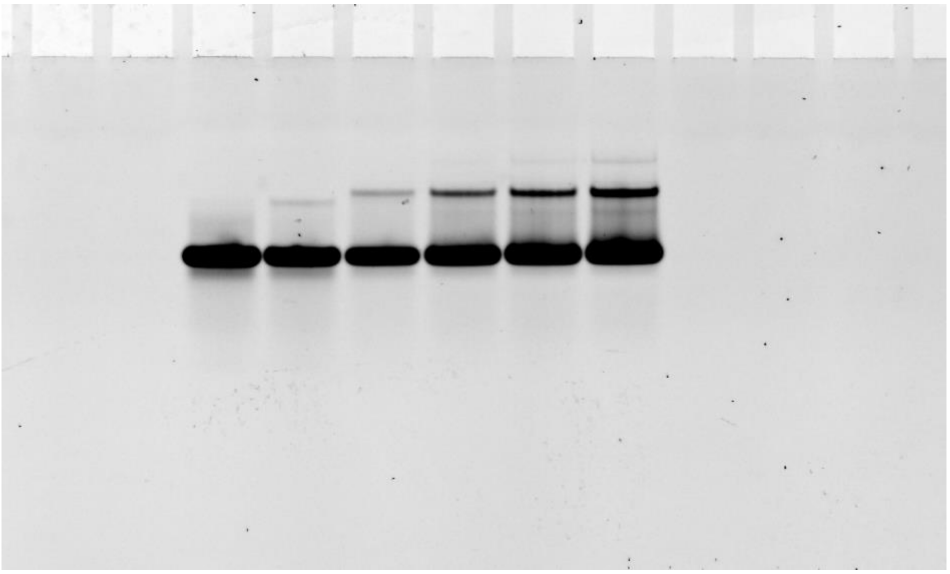

Supplementary Figure 6: Uncropped image of Figure 3C.

Supplementary Tables:

Supplementary Table 1. Data collection and refinement statistics (molecular replacement)

**Data collection**	
Space group	C 1 2 1
Cell dimensions	
a, *b*, *c* (Å)	114.82, 48.41, 139.84
α, β, γ (°)	90, 90, 90
Resolution (Å)	69.92-2.69 (2.79-2.69) *
R_{sym} or R_{merge}	0.1147 (0.5649)
$I / \sigma I$	2.16 (0.57)
Completeness (%)	97.54 (83.55)
Redundancy	2.0 (2.0)
**Refinement**	
Resolution (Å)	69.92-2.69
No. reflections	42346
R_{work} / R_{free}	0.2528/0.2937
No. atoms	4941
Macromolecules	4882
Ligand/ion	21
Water	38
B-factors	
Macromolecules	71.28
Ligand/ion	83.52
Water	65.19
R.m.s. deviations	
Bond lengths (Å)	0.007
Bond angles (°)	0.92

*Values in parentheses are for highest-resolution shell.

Supplementary Table 2: List of all oligonucleotide sequences used in this study.

Oligo Name	Sequence
10-23 Xstal mA	GCTGGGATmACATTGTGCGAAAGCACAATGGGCTAGCTACAACGAATCCCAGC
10-23 Xstal rA	GCTGGGArUrArCAATCCTAGTTATAGGATTGGGCTAGCTACAACGAATCCCAGC
FAM10-23 DNAzyme	FAM-CTCGCAGTATAGGCTAGCTACAACGAATCGCATAGG
10-23 Substrate mA	CCTATGCGAUmACATACTGCGAG
10-23 SnapBack	TATCCGCGGAGACGCGGATAGGGCTAGCTACAACGAAGCCTATCGGAGACGATAGGC
10-23 DNAzyme	CTCGCAGTATAGGCTAGCTACAACGAATCGCATAGG
FAM 10-23 substrate	FAM-CCTATGCGArUrArCATACTGCGAG
